# P-893. Comparative Antibiotic Stewardship Practices at Upper-Income versus Lower-Income Hospitals in Southern Malawi

**DOI:** 10.1093/ofid/ofaf695.1101

**Published:** 2026-01-11

**Authors:** Hannah Moody, Paddington Mbumbgwa, Akuzike Makondesa, Mphatso Mafunga, Matthew Cappiello, Melissa Pender

**Affiliations:** Loma Linda University, Loma Linda, California; Loma Linda University School of Medicine, Loma Linda, California; Malamulo Adventist Hospital, Blantyre, Thyolo, Malawi; Malamulo Adventist Hospital, Blantyre, Thyolo, Malawi; Loma Linda University, Loma Linda, California; Malamulo Adventist Hospital, Blantyre, Thyolo, Malawi

## Abstract

**Background:**

Malawi suffers from high rates of antimicrobial resistance, with a Drug Resistance Index currently estimated higher than India and Argentina. Stewardship literature in sub-Saharan Africa shows positive impact of regional stewardship programs,though significant variation is seen in control populations and clinical and microbiologic outcomes across study sites.

**Table 1:**
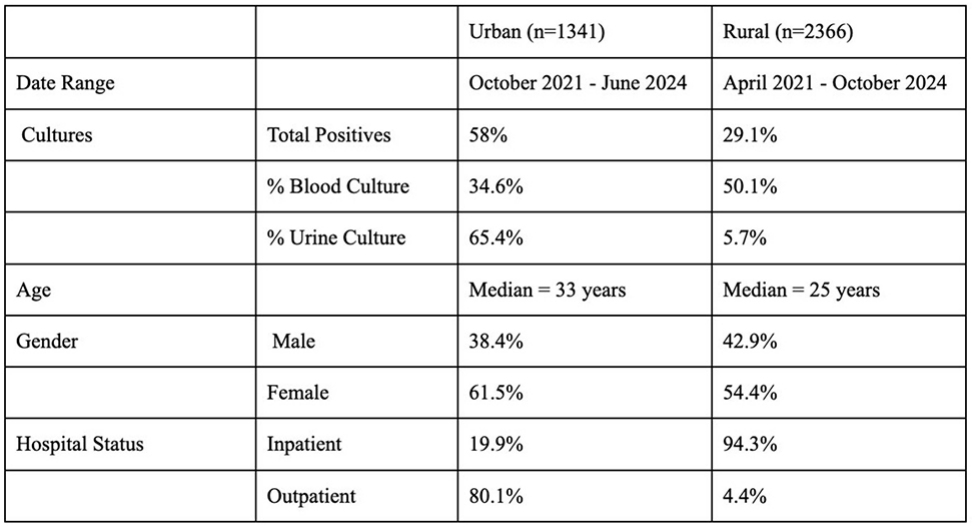
Descriptive Statistics

**Table 2:**
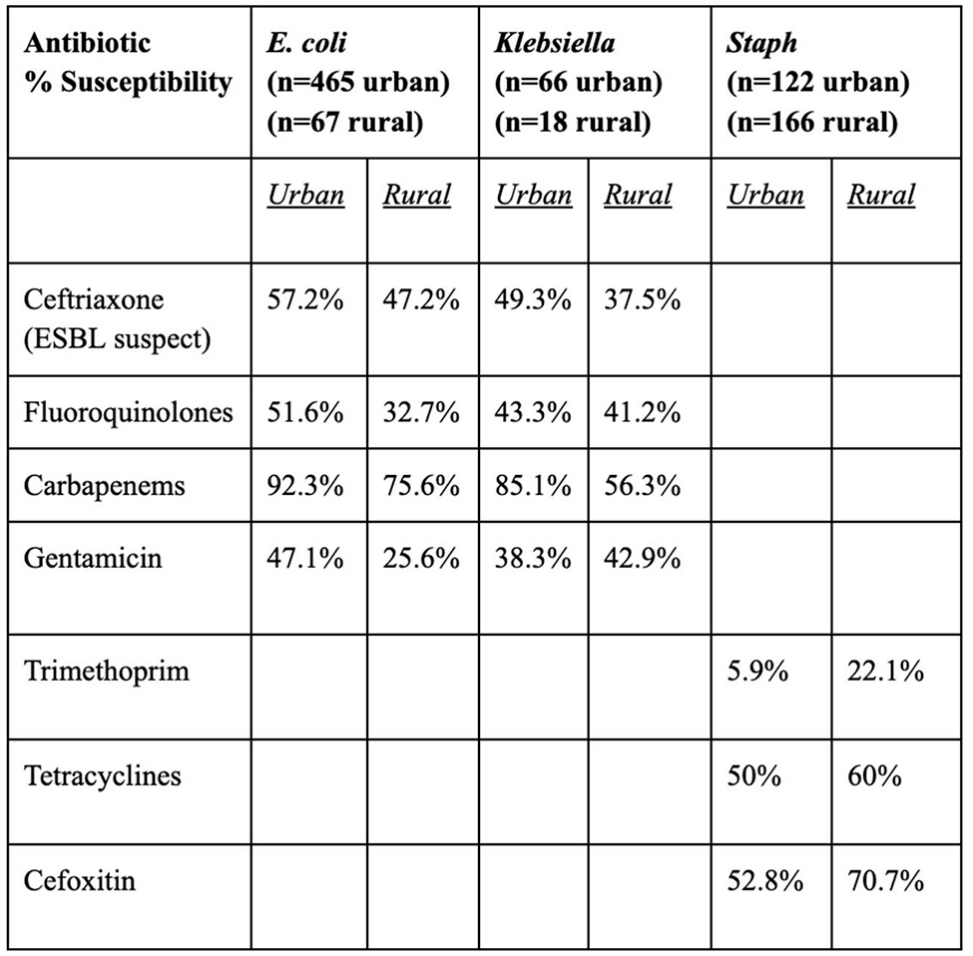
Comparative Antimicrobial Resistance at Urban and Rural Sites

**Methods:**

Clinical antibiograms and provider prescribing data were compared between an urban upper-income referral hospital in Blantyre District, southern Malawi (n=1341) and a rural safety-net hospital in Thyolo District, southern Malawi (n=2366). (Table 1)

**Table 3:**
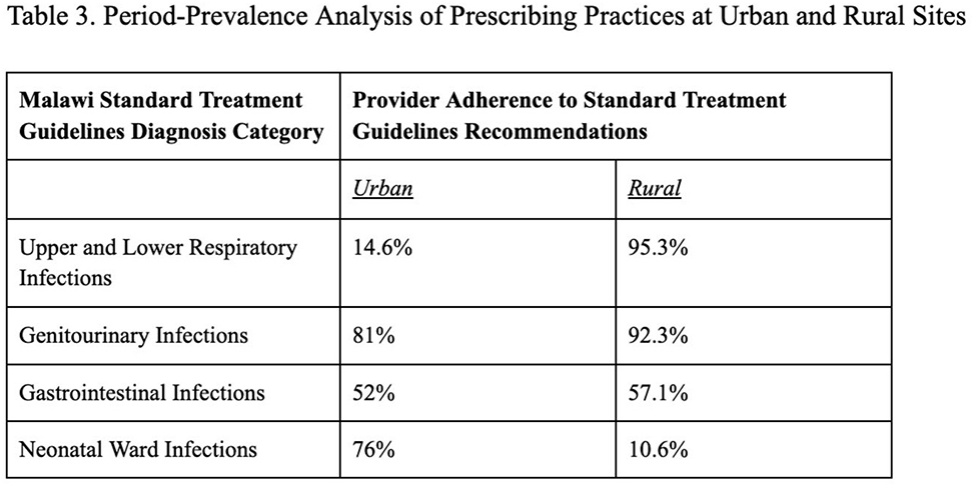
Period Prevalence Analysis of Prescribing Practices at Urban and Rural Sites

**Table 4:**
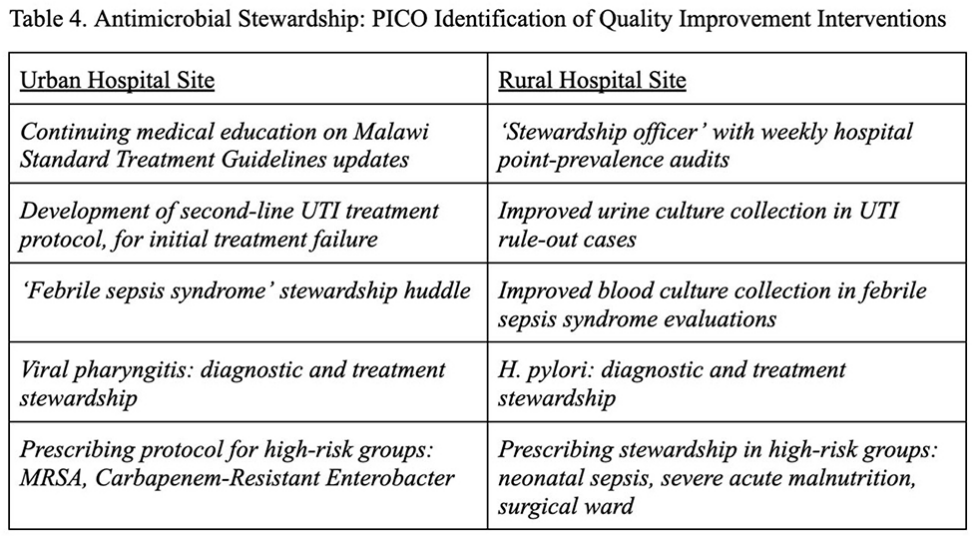
Antimicrobial Stewardship: PICO Identification of Quality Improvement Interventions

**Results:**

High rates of ESBL-suspect ceftriaxone-resistant gram negatives were seen at both sites (42.8% versus 52.8% E. coli, 50.7% versus 62.5% Klebsiella) as well as high rates of methicillin-resistant *Staph aureus* (47.2% versus 29.3%) (Table 2). Despite a substantially lower rate of carbapenem scripts at the rural safety-net site, a higher rate of carbapenem resistance was seen at the rural-safety net site (24.4% E. coli, 43.7% Klebsiella) as compared to the urban upper-income site (7.7% E. coli, 14.9% Klebsiella). Adverse outcomes were associated with antimicrobial resistance in urban and rural settings, including hospital cost (p< 0.01) and patient death (p< 0.01) in the urban cohort as well as length of stay (p< 0.01) in the rural cohort. Rural safety-net providers were overall more adherent to Malawi Standard Treatment Guidelines than urban upper-income providers (Table 3), although rural challenges were noted in subspecialty ward subgroups including neonatology. Additionally, unique local challenges at each hospital (Table 4) were identified as areas for stewardship improvement.

**Conclusion:**

Data suggests that stewardship approaches are not one-size-fits-all in southern Malawi, and need to be tailored to unique local and institutional needs.

**Disclosures:**

All Authors: No reported disclosures

